# Lymphocyte infiltration in breast cancer: A promising prognostic indicator

**DOI:** 10.1097/MD.0000000000040845

**Published:** 2024-12-06

**Authors:** Emmanuel Ifeanyi Obeagu, Getrude Uzoma Obeagu

**Affiliations:** a Department of Medical Laboratory Science, Kampala International University, Ishaka, Uganda; b School of Nursing Science, Kampala International University, Ishaka, Uganda.

**Keywords:** breast cancer, immunotherapy, prognosis, tumor microenvironment, tumor-infiltrating lymphocytes (TILs)

## Abstract

Breast cancer is a leading cause of cancer-related mortality among women worldwide, necessitating the identification of reliable prognostic markers to guide treatment and improve patient outcomes. Recent research has highlighted the prognostic significance of tumor-infiltrating lymphocytes (TILs) in breast cancer, with high levels of TILs being associated with improved survival rates and better responses to therapy. This review delves into the mechanisms driving lymphocyte infiltration, its clinical implications, and the potential for TILs to serve as predictive biomarkers in breast cancer management. The presence of TILs within the tumor microenvironment reflects a dynamic interplay between tumor cells and the host immune system. Chemokine signaling, antigen presentation, and immune checkpoint interactions are key mechanisms that facilitate the recruitment and activity of lymphocytes at the tumor site. Clinically, the density of TILs varies across breast cancer subtypes, with the most significant prognostic value observed in triple-negative and HER2-positive breast cancers. High TIL levels correlate with improved overall survival and disease-free survival, underscoring their potential as a valuable prognostic indicator. Therapeutically, the role of TILs has opened new avenues in breast cancer treatment, particularly in the realm of immunotherapy. Immune checkpoint inhibitors, adoptive cell therapy, and combination therapies leveraging TILs are being explored to enhance antitumor responses. As research progresses, the integration of TIL assessment into routine clinical practice could revolutionize personalized treatment strategies, ultimately improving prognostic accuracy and patient outcomes in breast cancer care.

## 1. Introduction

Breast cancer is one of the most prevalent cancers among women globally and remains a significant cause of cancer-related mortality.^[1]^ Despite advances in early detection and treatment, the heterogeneity of breast cancer poses challenges in predicting clinical outcomes and tailoring effective therapies. Traditionally, prognostic indicators such as tumor size, grade, lymph node involvement, and hormone receptor status have been utilized to guide clinical decisions.^[2]^ However, these factors alone often fall short in providing a comprehensive prognosis, highlighting the need for additional reliable biomarkers. Recent research has brought attention to the immune system’s role in cancer progression and response to treatment, particularly the presence of tumor-infiltrating lymphocytes (TILs) within the tumor microenvironment (TME).^[3]^ TILs are a heterogeneous population of immune cells, predominantly comprising T cells, but also including B cells and natural killer (NK) cells. Their infiltration into tumors indicates an active immune response against the malignancy, which can have significant prognostic implications.^[4]^ The concept of immune surveillance, where the immune system recognizes and eliminates emerging tumor cells, is foundational for understanding the role of TILs.^[5]^ Tumors, however, can develop mechanisms to evade immune detection and destruction. The presence of TILs within a tumor signifies an ongoing battle between the immune system and cancer cells. This dynamic interaction can influence tumor behavior and patient outcomes, making TILs a focal point in cancer research. Mechanisms driving lymphocyte infiltration are complex and involve multiple factors.^[6]^ Chemokine signaling plays a critical role, with tumor cells secreting chemokines that attract lymphocytes to the tumor site. Additionally, the presentation of tumor antigens by major histocompatibility complex molecules on cancer cells can trigger an immune response, leading to lymphocyte infiltration. Moreover, immune checkpoint pathways, such as the programmed death-1 (PD-1)/programmed death-ligand 1 (PD-L1) axis, can modulate T cell activity, impacting the extent and effectiveness of lymphocyte infiltration.^[6]^

Clinically, the prognostic value of TILs has been most prominently observed in certain breast cancer subtypes. In triple-negative breast cancer and HER2-positive breast cancer, high levels of TILs have been correlated with better overall survival (OS) and disease-free survival (DFS).^[7]^ This correlation underscores the potential of TILs as a prognostic biomarker, particularly in aggressive breast cancer subtypes where traditional markers may be less predictive. The TME significantly influences the behavior and efficacy of TILs.^[8]^ A favorable TME, characterized by the presence of active immune cells and a lack of immunosuppressive factors, supports robust antitumor immunity. Conversely, an immunosuppressive TME can inhibit lymphocyte function and promote tumor progression. Gene expression profiles indicative of immune activation, protein markers such as PD-L1, and immunohistochemical (IHC) assessment of TIL levels are among the methods being explored.^[9]^ These biomarkers can provide insights into the immune landscape of tumors and help predict patient response to various treatments, including immunotherapy. The therapeutic implications of TILs in breast cancer are profound, especially in the context of immunotherapy. Immune checkpoint inhibitors targeting pathways like PD-1/PD-L1 and CTLA-4 have shown promise in enhancing T cell activity against tumors.^[10]^ Additionally, adoptive cell therapy, where TILs are extracted, expanded ex vivo, and reinfused into the patient, represents a novel approach to boosting the immune response. Combination therapies that integrate immunotherapy with conventional treatments such as chemotherapy and radiation are also being investigated for their synergistic effects.^[10]^

Breast cancer, as a highly heterogeneous disease, poses significant challenges in prognostication and treatment. Traditional prognostic factors, while useful, often do not fully capture the complexities of tumor biology and the diverse clinical outcomes observed in patients. This necessitates the exploration of additional biomarkers that can provide deeper insights into tumor behavior and patient prognosis. The immune system’s role in cancer progression and therapy response has garnered considerable interest in recent years. Among the various components of the immune system, TILs have emerged as a particularly promising prognostic indicator. TILs reflect the host immune response to the tumor and their presence is often associated with improved clinical outcomes. This relationship underscores the potential of TILs to serve not only as a prognostic marker but also as a therapeutic target. Understanding the mechanisms driving lymphocyte infiltration into tumors, the influence of the TME, and the interaction between TILs and cancer cells can provide valuable insights into tumor immunology. Moreover, the ability to predict TIL levels and their functional state through biomarkers can enhance the precision of prognostic assessments and guide treatment decisions. As immunotherapy continues to advance, harnessing the prognostic and therapeutic potential of TILs could revolutionize breast cancer care. This review aims to consolidate current knowledge on TILs in breast cancer, highlighting their prognostic value and exploring the future directions for research and clinical application.^[8–10]^

## 2. Aim

The aim of this review is to comprehensively explore the role of lymphocyte infiltration in breast cancer as a prognostic indicator.

## 3. Review methodology

### 3.1. Literature search strategy

A comprehensive literature search was conducted to gather relevant studies and reviews on the role of lymphocyte infiltration in breast cancer. The search was performed using several databases including PubMed, Scopus, Web of Science, and Google Scholar. Keywords and phrases used in the search included “breast cancer,” “tumor-infiltrating lymphocytes,” “TILs,” “prognostic indicator,” “immune infiltration,” “tumor microenvironment,” “immune checkpoint,” “immunotherapy,” and “clinical outcomes.”

### 3.2. Inclusion and exclusion criteria

To ensure the relevance and quality of the included studies, the following inclusion and exclusion criteria were applied:

- Inclusion criteria:
- Peer-reviewed articles published in English.
- Studies focusing on the role of TILs in breast cancer.
- Research articles, reviews, and meta-analyses that provide data on the prognostic significance, mechanisms, and therapeutic implications of TILs.
- Studies involving human subjects and clinical data.
- Exclusion criteria:
- Articles not available in English.
- Studies with insufficient or unclear data regarding TILs in breast cancer.
- Non-peer-reviewed sources, such as conference abstracts, editorials, and opinion pieces.

### 3.3. Data extraction

Data extraction was performed systematically to gather information on the following aspects:

- Study characteristics: author(s), publication year, study design, and sample size.
- Patient demographics and clinical characteristics.
- Methods used to assess TILs, including IHC, gene expression profiling, and other molecular techniques.
- Main findings related to the presence and density of TILs and their correlation with clinical outcomes (e.g., OS, DFS).
- Mechanistic insights into lymphocyte infiltration and the role of the TME.
- Predictive biomarkers for TIL presence and activity.
- Therapeutic approaches targeting TILs, including immune checkpoint inhibitors and adoptive cell therapy.

### 3.4. Quality assessment

The quality of the included studies was assessed using standard criteria for evaluating research validity and reliability. This included examining the study design, sample size, methodological rigor, and the clarity and consistency of reported results. Studies were categorized based on the level of evidence, ranging from high-quality randomized controlled trials to observational studies and case series.

### 3.5. Lymphocyte subtypes in breast cancer

Each subtype plays a distinct role in modulating the immune response against cancer cells, and their presence or absence can significantly impact patient prognosis and response to therapy.

T cells (CD3+): T cells are central to the adaptive immune response and are characterized by the expression of the CD3 marker. These cells can be further divided into 2 main subsets: cytotoxic T cells and helper T cells (Th cells).

Cytotoxic T cells (CD8+): these cells are the primary effector cells in the immune system’s attack against tumor cells. They recognize and kill cancer cells by identifying antigens presented by major histocompatibility complex class I molecules. The presence of a high number of CD8 + T cells in breast tumors has been associated with better clinical outcomes, as these cells directly target and eliminate tumor cells.^[11]^

Th cells (CD4+): these cells play a supportive role by secreting cytokines that enhance the function of other immune cells. They are crucial for orchestrating the overall immune response. Within this subset, there are further specializations, such as Th1 cells that promote cellular immunity and Th2 cells that support humoral immunity. The balance between different helper T cell subsets can influence the effectiveness of the antitumor response.^[12]^

B cells (CD20+): B cells are an essential component of the adaptive immune system and are identified by the CD20 marker. They produce antibodies against tumor antigens, facilitating the identification and destruction of cancer cells. Additionally, B cells can act as antigen-presenting cells to activate T cells and secrete cytokines that modulate the immune response. The role of B cells in breast cancer is complex, as they can have both pro-tumor and antitumor effects depending on the context of the TME.^[13]^

Regulatory T cells (Tregs) (CD4 + CD25 + FOXP3+): Tregs are a subset of T cells that express the markers CD4, CD25, and FOXP3. They play a crucial role in maintaining immune homeostasis by suppressing excessive immune responses and preventing autoimmunity. However, in the context of cancer, Tregs can be detrimental as they inhibit the antitumor immune response, allowing cancer cells to evade immune surveillance. High levels of Tregs in breast cancer are often associated with poorer prognosis.^[14]^

NK cells (CD56+): NK cells are part of the innate immune system and are characterized by the expression of the CD56 marker. They can kill tumor cells without prior sensitization by recognizing stress-induced ligands on the surface of cancer cells. NK cells play a critical role in the early defense against tumors and can influence the adaptive immune response. Their presence in breast tumors has been associated with better clinical outcomes.^[13]^

Memory T cells (CD45RO+): memory T cells are a subset of T cells that express the CD45RO marker. They provide long-term immunity by quickly responding to previously encountered antigens. Memory T cells are crucial for sustained antitumor immunity and can help prevent cancer recurrence by maintaining a vigilant immune surveillance.^[14]^

### 3.6. The prognostic significance of lymphocyte infiltration in breast cancer

The prognostic significance of lymphocyte infiltration in breast cancer is a topic of growing interest and research within the field of oncology. Lymphocyte infiltration, referring to the presence of immune cells, particularly lymphocytes, within the TME, has been recognized as a valuable prognostic indicator in breast cancer. Here, we discuss the significance of lymphocyte infiltration and its implications for predicting disease outcomes and guiding clinical decision-making:

(1) Association with OS: numerous studies have demonstrated a positive correlation between increased lymphocyte infiltration and improved OS in breast cancer patients. Higher levels of TILs are often associated with a more favorable prognosis.^[15]^ This suggests that the immune system’s ability to recognize and combat cancer cells within the TME can be a powerful indicator of patient outcomes.
(2) Correlation with DFS: lymphocyte infiltration has also shown a strong association with DFS, indicating that patients with a greater presence of lymphocytes in their tumors tend to experience longer periods without cancer recurrence.^[16]^ This relationship underscores the potential clinical relevance of assessing lymphocyte infiltration when determining patient prognosis and treatment strategies.
(3) Predictive value in different breast cancer subtypes: lymphocyte infiltration’s prognostic significance may vary across different breast cancer subtypes.^[17]^ For instance, triple-negative breast cancer and HER2-positive breast cancer often exhibit higher levels of TILs and may benefit more from immune-based therapies. In contrast, hormone receptor-positive breast cancer may exhibit a more complex relationship with lymphocyte infiltration.
(4) Impact of lymphocyte subtypes on prognosis: the specific subtypes of lymphocytes present within the TME can further influence prognostic outcomes.^[18]^ For example, an abundance of cytotoxic T lymphocytes (CTLs) may be particularly favorable, as they directly target and kill cancer cells. In contrast, an excess of Tregs, which can suppress immune responses, may be associated with poorer prognosis.
(5) Immunotherapy implications: lymphocyte infiltration has significant implications for the use of immunotherapeutic strategies in breast cancer. Patients with high levels of TILs may be more responsive to immune checkpoint inhibitors and other immunotherapies. Assessing lymphocyte infiltration can guide treatment decisions, identifying those who are likely to benefit from immunotherapy.^[19]^
(6) Influence on treatment decisions: in clinical practice, the assessment of lymphocyte infiltration can help oncologists tailor treatment plans. Patients with a favorable lymphocyte profile may be candidates for less aggressive therapy, while those with lower lymphocyte infiltration may require more intensive treatments.

### 3.7. Mechanisms of lymphocyte-mediated antitumor activity in breast cancer

Lymphocytes play a critical role in mediating antitumor activity in breast cancer by employing various mechanisms to recognize, target, and destroy cancer cells within the TME. Understanding these mechanisms is essential for developing effective therapeutic strategies. Here are the key mechanisms through which lymphocytes combat breast cancer:

(1) Antigen recognition: lymphocytes, particularly T cells (both CTLs and Th cells), recognize cancer cells by identifying specific antigens displayed on the surface of these cells.^[20]^ Antigen-presenting cells, such as dendritic cells, process tumor antigens and present them to T cells, initiating an immune response.
(2) Cytotoxicity: CTLs are the primary effectors of antitumor immunity. CTLs can directly kill cancer cells by releasing cytotoxic molecules, such as perforin and granzymes, which induce apoptosis (cell death) in the target cells. This process is often referred to as cell-mediated cytotoxicity.
(3) Th cell activation: Th cells play a critical role in supporting the immune response. They help activate CTLs and other immune cells, enhancing their antitumor activity. Th cells secrete cytokines and provide the necessary signals to coordinate an effective immune response against cancer.
(4) Immunological memory: lymphocytes, particularly memory T cells, can develop immunological memory after encountering cancer cells. This means that if the cancer returns, the immune system can mount a faster and more effective response, potentially preventing cancer recurrence.^[21]^
(5) Immune checkpoint regulation: immune checkpoint molecules, such as PD-1 and CTLA-4, regulate the intensity of the immune response.^[22]^ In breast cancer and other malignancies, cancer cells can exploit these checkpoints to evade immune surveillance. Immune checkpoint inhibitors, such as anti-PD-1 and anti-CTLA-4 antibodies, can be used to block these inhibitory signals, reactivating lymphocyte-mediated antitumor activity.
(6) Induction of apoptosis: lymphocytes, through various mechanisms, induce programmed cell death (apoptosis) in cancer cells. This process is a crucial part of the immune response against tumors, as it eliminates malignant cells while minimizing collateral damage to healthy tissue.^[23]^
(7) Cytokine production: lymphocytes produce cytokines, such as interferons and interleukins, which modulate the immune response. These cytokines can enhance the activity of other immune cells and promote an antitumor environment within the TME.^[24]^
(8) Recruitment of other immune cells: lymphocytes can recruit other immune cells, including macrophages and NK cells, to collaborate in the antitumor response.^[25]^ This collaborative effort can amplify the immune attack against cancer cells.
(9) Tertiary lymphoid structures (TLS): in some cases, lymphocytes can organize themselves into TLS within the tumor. TLS can serve as centers for immune cell activation and interaction, potentially enhancing the immune response against breast cancer.

### 3.8. Clinical assessment of lymphocyte infiltration in breast cancer

The clinical assessment of lymphocyte infiltration in breast cancer involves various methods and techniques to evaluate the presence and activity of lymphocytes within the TME. Accurate assessment of lymphocyte infiltration is crucial for understanding the patient’s immune response, predicting disease outcomes, and guiding treatment decisions. Here are some of the key methods used for clinical assessment:

(1) Histopathological evaluation: pathologists examine breast cancer tissue samples obtained through biopsies or surgical resections. They assess the degree of lymphocyte infiltration by visually examining the tissue under a microscope. This evaluation often involves scoring systems, such as the immune cell or stromal tumor-infiltrating lymphocyte score, to quantify the extent of lymphocytes within the tumor and the surrounding stroma.^[26–28]^
(2) IHC: immunohistochemistry is a technique that uses specific antibodies to stain and identify different immune cell populations within the tumor tissue. Antibodies targeting markers like CD3 (pan-T cell marker), CD8 (cytotoxic T cell marker), CD20 (B cell marker), and CD56 (NK cell marker) are commonly used to identify and quantify lymphocytes in the TME.^[29]^
(3) Flow cytometry: flow cytometry is a laboratory technique that allows for the quantification and characterization of lymphocyte subtypes in breast cancer tissue. It involves disaggregating the tissue into single-cell suspensions and using fluorescently labeled antibodies to identify and quantify various immune cell populations. Flow cytometry provides a detailed analysis of lymphocyte subsets and their activation status.
(4) Genomic and transcriptomic profiling: genomic and transcriptomic profiling techniques, such as RNA sequencing, can provide insights into the presence and functional activity of lymphocytes. These methods assess the expression of immune-related genes and cytokines, which can indicate the immune response within the tumor.^[30]^
(5) Multiplex immunofluorescence: this advanced technique allows for the simultaneous assessment of multiple immune cell markers in tissue samples. Multiplex immunofluorescence can provide a detailed spatial analysis of lymphocyte distribution within the tumor and its microenvironment.
(6) Molecular imaging: molecular imaging techniques, such as positron emission tomography and single-photon emission computed tomography, can be used to visualize immune cell activity within the tumor using radiolabeled tracers. These techniques provide functional information about lymphocyte infiltration and activation.^[31]^
(7) Artificial intelligence: artificial intelligence involves scanning and digitizing histopathological slides, enabling computer-assisted analysis. Machine learning algorithms and artificial intelligence can be applied to assess lymphocyte infiltration patterns, providing objective and quantitative data.
(8) TLS: the presence of TLS within the tumor can be evaluated through histopathological examination and immunohistochemistry. The formation of TLS can indicate an organized immune response within the TME.^[32]^

### 3.9. Lymphocyte infiltration and breast cancer treatment

Lymphocyte infiltration in breast cancer is a topic of increasing significance in the context of treatment strategies. The presence and activity of lymphocytes within the TME can have profound implications for how breast cancer is managed and treated. Lymphocyte infiltration is of particular relevance in the era of immunotherapy. Immunotherapeutic approaches, such as immune checkpoint inhibitors (e.g., anti-PD-1 and anti-CTLA-4 antibodies), aim to enhance the immune system’s response against cancer. Patients with high levels of lymphocyte infiltration, especially CTLs, are more likely to benefit from these therapies. Immunotherapy can help “unleash” the immune system to target and destroy cancer cells more effectively.^[33–36]^ The assessment of lymphocyte infiltration is used to predict a patient’s response to immunotherapy. High levels of TILs and a favorable lymphocyte profile often correlate with better responses to immune checkpoint inhibitors. In some cases, immunotherapy is considered for breast cancer patients who exhibit strong lymphocyte infiltration, as they are more likely to experience clinical benefits. Lymphocytes can also influence the response to conventional chemotherapy and targeted therapies.^[37]^ An immune-rich microenvironment can enhance the effectiveness of chemotherapy. For instance, some chemotherapy drugs work synergistically with the immune system to kill cancer cells. Additionally, targeted therapies, such as HER2-targeted treatments for HER2-positive breast cancer, may have better outcomes when combined with an active immune response.

The assessment of lymphocyte infiltration can help guide personalized treatment decisions. Patients with a strong lymphocyte presence may be candidates for less aggressive treatments, minimizing the potential for unnecessary side effects.^[38]^ Conversely, patients with lower lymphocyte infiltration may require more intensive therapeutic approaches to ensure effective tumor control. Researchers are exploring combination therapies that leverage both traditional treatments (e.g., surgery, chemotherapy, radiation) and immunotherapies to enhance the antitumor immune response. The interplay between lymphocytes and these treatment modalities is a critical aspect of designing effective combination regimens. Assessing lymphocyte infiltration before, during, and after treatment can provide insights into treatment efficacy.^[39]^ Changes in lymphocyte infiltration levels during therapy may indicate the patient’s response to treatment and can guide adjustments to the treatment plan. While lymphocyte infiltration is often associated with positive treatment outcomes, some breast cancers can develop mechanisms to evade the immune response, leading to immunotherapy resistance. Lymphocyte infiltration plays a central role in the development and optimization of breast cancer treatment strategies. It affects patient responsiveness to a variety of treatments, particularly immunotherapy, and guides personalized therapy decisions. As our understanding of the immune landscape in breast cancer continues to evolve, leveraging lymphocyte infiltration as a tool for enhancing treatment outcomes remains a promising avenue for improving patient care.^[34–37]^

Lymphocyte infiltration has significant clinical implications for breast cancer diagnosis, prognosis, and treatment. The density and composition of lymphocyte subsets within tumors are often used as biomarkers for assessing disease progression and predicting patient outcomes. High levels of TILs, particularly CD8 + T cells, are associated with favorable clinical outcomes in breast cancer. Elevated TILs correlate with improved OS and DFS, particularly in aggressive breast cancer subtypes such as triple-negative and HER2-positive breast cancers. The presence of these cells indicates a robust antitumor immune response, which is often linked to a better prognosis. Lymphocyte infiltration can also serve as a predictive biomarker for treatment response. High TIL levels are associated with increased sensitivity to chemotherapy and targeted therapies. For instance, breast cancer patients with high TIL levels often show better responses to neoadjuvant chemotherapy, which can lead to higher rates of pathologic complete response. In HER2-positive breast cancer, high levels of TILs are associated with improved outcomes following treatment with trastuzumab, an anti-HER2 targeted therapy.^[33–35]^

Harnessing the immune system to fight breast cancer has led to the development of several innovative therapeutic strategies. These approaches aim to enhance the immune response against tumor cells, leveraging the role of lymphocytes in cancer treatment. Immune checkpoint inhibitors are a class of drugs designed to block immune checkpoint proteins that cancer cells use to evade immune surveillance. PD-1 and PD-L1 inhibitors, such as pembrolizumab and atezolizumab, have shown promise in treating breast cancer, especially in patients with high TIL levels.^[36,37]^ By inhibiting the interaction between PD-1 and PD-L1, these drugs can reinvigorate exhausted T cells, enhancing their ability to recognize and attack cancer cells. Cancer vaccines aim to stimulate the immune system to target tumor-specific antigens. These vaccines can be designed to elicit a strong immune response against cancer cells, potentially increasing lymphocyte infiltration into the tumor. Examples include HER2-targeted vaccines and peptide-based vaccines that aim to provoke a specific immune response against tumor-associated antigens. Adoptive cell therapy involves the expansion and reinfusion of patient-derived immune cells to enhance the antitumor immune response. Two prominent approaches in this area are TIL therapy and chimeric antigen receptor (CAR) T-cell therapy. In TIL therapy, lymphocytes are extracted from the tumor, expanded ex vivo, and then infused back into the patient to boost the antitumor immune response. CAR T-cell therapy involves engineering patients’ T cells to express a receptor specific for tumor antigens, such as HER2, enabling them to target and destroy cancer cells more effectively. Monoclonal antibodies, such as trastuzumab for HER2-positive breast cancer, are designed to target specific antigens on tumor cells. These antibodies can induce direct tumor cell killing, mediate ADCC, and enhance the immune system’s ability to recognize and destroy cancer cells. Combining monoclonal antibodies with other therapies, such as chemotherapy or immune checkpoint inhibitors, is being explored to improve treatment outcomes. Lymphocyte infiltration in breast cancer represents a critical aspect of the immune response to tumors and has significant implications for both prognosis and treatment. The presence and activity of various lymphocyte subtypes, such as cytotoxic T cells, B cells, Tregs, NK cells, and memory T cells, provide valuable insights into the immune landscape of breast cancer.^[38–41]^

## 4. Challenges and future directions of lymphocyte infiltration and breast cancer

Challenges and future directions in the study of lymphocyte infiltration in breast cancer are crucial to advancing our understanding of this dynamic interaction and improving patient outcomes. Several challenges and promising areas of research and clinical development are as follows.

### 4.1. Challenges

Breast cancer is a highly heterogeneous disease, and this heterogeneity extends to the immune microenvironment. Different subtypes of breast cancer can exhibit variations in the extent and composition of lymphocyte infiltration, posing challenges for standardizing assessment and treatment approaches.^[40]^ Tumors can employ various immunosuppressive mechanisms to evade the immune response, limiting the effectiveness of lymphocyte-mediated antitumor activity. Identifying and targeting these mechanisms is an ongoing challenge in breast cancer immunotherapy.^[41]^ Some breast cancers may develop resistance to immunotherapy over time, necessitating research into the mechanisms behind this resistance and the development of strategies to overcome it.^[26]^ While the presence of lymphocytes is generally associated with positive outcomes, treatment strategies need to be personalized. Patient-specific factors, including the type and location of lymphocytes within the tumor, must be considered when making treatment decisions. Establishing standardized methods for assessing lymphocyte infiltration is critical. There is a need for consistent scoring systems and diagnostic criteria to ensure that lymphocyte assessment is reliable and comparable across different clinical settings.

### 4.2. Implications of lymphocyte infiltration for breast cancer treatment

Lymphocyte infiltration into breast tumors reveals a great deal about the immune response to cancer and has profound implications for both current and future breast cancer treatments. This section explores how understanding lymphocyte subtypes can guide therapeutic strategies, influence treatment decisions, and shape the future of breast cancer care.

#### 4.2.1. Personalized treatment strategies

One of the most significant implications of lymphocyte infiltration for breast cancer treatment is the potential for personalized medicine. By assessing the presence and types of lymphocytes in a patient’s tumor, clinicians can tailor treatments to the specific immune environment of the cancer. This personalized approach aims to maximize therapeutic efficacy and minimize side effects. High levels of TILs, particularly CD8 + cytotoxic T cells and NK cells, often correlate with better prognosis and increased response to therapies. Measuring TIL levels can help identify patients who are more likely to benefit from immunotherapies or certain chemotherapy regimens. For instance, patients with high CD8 + T cell counts might be considered for trials of immune checkpoint inhibitors or combination therapies that exploit their existing antitumor immunity. For patients with high TIL levels and a good prognosis, clinicians might opt for less aggressive adjuvant therapies, focusing on minimizing treatment-related toxicities. Conversely, for patients with low TIL levels, more intensive treatments might be required to overcome a less robust immune response.^[41]^

#### 4.2.2. Enhanced immunotherapy approaches

The presence of specific lymphocyte subtypes provides valuable insights into how immunotherapies can be designed and applied in breast cancer treatment. Advances in this field are focused on harnessing the immune system’s power to fight cancer. Immune checkpoint inhibitors such as PD-1/PD-L1 blockers (e.g., pembrolizumab, atezolizumab) have shown efficacy in treating cancers with high levels of TILs. In breast cancer, these drugs are often used in cases where high TIL levels indicate that the immune system is already primed to attack the tumor. Ongoing research aims to refine these therapies to improve their effectiveness for patients with low TIL levels or to identify biomarkers that predict which patients will benefit most from these treatments. CAR T-cell therapy involves engineering a patient’s T cells to express a receptor that targets cancer cells. This approach has been successful in hematological malignancies and is now being adapted for solid tumors like breast cancer. Researchers are exploring CAR T-cells targeting HER2 and other tumor-associated antigens, guided by the understanding that high levels of TILs might predict better responses to such therapies. Cancer vaccines aim to stimulate the immune system against tumor-specific antigens. The development of vaccines targeting HER2, tumor-associated antigens, or neoantigens is informed by knowledge of lymphocyte dynamics in tumors. High levels of TILs might indicate that a patient’s immune system is already engaged against the tumor, making vaccines a potential way to further boost this response.^[26]^

#### 4.2.3. Optimizing chemotherapy and targeted therapies

The effectiveness of chemotherapy can be influenced by the tumor’s immune environment. For instance, high TIL levels are associated with better responses to neoadjuvant chemotherapy, which is used before surgery to shrink tumors. This insight can guide the selection of chemotherapy regimens and inform decisions about the need for additional treatments based on the tumor’s immune profile. Targeted therapies, such as HER2-targeted treatments (e.g., trastuzumab), can be combined with other therapies based on the lymphocyte composition of the tumor. For example, in HER2-positive breast cancer, the presence of high TILs may enhance the effectiveness of trastuzumab, and ongoing research explores combining targeted therapies with immunotherapy to further improve outcomes.^[42]^

#### 4.2.4. Developing new therapeutic modalities

The study of lymphocyte infiltration has spurred the development of new therapeutic modalities aimed at enhancing the immune response against breast cancer. These novel agents are designed to bind simultaneously to T cells and cancer cells, bridging them for a more effective immune attack. Bi-specific antibodies like those targeting HER2 and CD3 are being explored in clinical trials, with the goal of harnessing the existing antitumor activity suggested by TIL profiles. This innovative approach uses genetically modified viruses to selectively infect and kill cancer cells. The success of oncolytic virus therapy is often enhanced by the immune environment of the tumor. Research is ongoing to understand how TILs can be leveraged to improve the efficacy of these treatments. Techniques such as TIL therapy and CAR T-cell therapy are being expanded to include new targets and optimize cell engineering processes. The presence of TILs in the tumor is used to guide the selection of cells for expansion and treatment, aiming to maximize therapeutic benefits.^[43]^

#### 4.2.5. Predicting and overcoming therapy resistance

A deep understanding of lymphocyte subtypes can also help in predicting and overcoming therapy resistance. Research into the interactions between TILs and tumor cells reveals mechanisms of immune evasion and resistance to therapy. For example, high levels of Tregs or immune checkpoints might contribute to therapy resistance. Identifying these mechanisms can lead to the development of strategies to overcome resistance, such as combining checkpoint inhibitors with therapies that deplete Tregs. Combining different therapeutic approaches can overcome resistance and improve patient outcomes. For example, combining immune checkpoint inhibitors with chemotherapy or targeted therapies might counteract mechanisms of resistance and provide a more comprehensive treatment strategy.^[44]^

#### 4.2.6. Guiding treatment decisions for metastatic breast cancer

In metastatic breast cancer, lymphocyte profiles help guide treatment decisions and strategies. Regular assessment of TIL levels and other lymphocyte markers can help monitor disease progression and treatment response in metastatic breast cancer. Changes in TIL levels might indicate how well the current treatment is working or whether adjustments are needed. In metastatic settings, lymphocyte profiles can inform decisions about the use of systemic therapies, such as chemotherapy, targeted therapies, or immunotherapies. For example, the presence of high TILs might support the use of immune-based therapies in addition to standard treatments.^[45]^

### 4.3. Future directions

Advancements in genomics and immunomics will lead to more precise immunotherapeutic strategies. Identifying patient-specific immune profiles and tumor characteristics will enable tailored immunotherapy regimens, maximizing therapeutic benefit while minimizing side effects.^[42]^ The future of breast cancer treatment may involve innovative combination therapies that leverage the synergy between immunotherapy and other treatment modalities. Optimizing the timing and sequencing of these therapies will be a focus of research. The discovery of specific biomarkers related to lymphocyte infiltration and immune response will enhance the accuracy of prognosis and treatment prediction. These biomarkers can be integrated into clinical practice for improved patient care. Exploring the interplay between the gut microbiome and the immune system could provide new avenues for enhancing lymphocyte-mediated antitumor responses. Strategies that manipulate the microbiome to improve immunotherapy outcomes are under investigation.^[43]^

CAR T-cell therapy and other cellular therapies have shown promise in other cancers and may be extended to breast cancer treatment. These therapies involve engineering immune cells to enhance their tumor-targeting capabilities. AI and machine learning will play an increasing role in analyzing complex datasets related to lymphocyte infiltration and treatment responses. These technologies can aid in identifying patterns and predictive factors in breast cancer. The future of breast cancer treatment will emphasize patient-centered care, taking into account individual preferences and values. Shared decision-making between patients and healthcare providers will be pivotal. Ongoing clinical trials are evaluating novel therapies and immunomodulators for breast cancer. Participation in well-designed clinical trials will be critical in advancing treatment options.^[44–46]^

## 5. Conclusion

Lymphocyte infiltration in breast cancer has emerged as a significant prognostic indicator, reflecting the intricate interplay between the tumor and the host immune system. High levels of TILs are associated with improved clinical outcomes, including better OS and DFS, particularly in aggressive breast cancer subtypes such as triple-negative and HER2-positive cancers. The presence of TILs signifies an active immune response against the tumor, making them a valuable biomarker for predicting prognosis and guiding therapeutic decisions. The TME plays a pivotal role in modulating TIL activity, with a favorable TME enhancing antitumor immunity and an immunosuppressive TME promoting tumor progression. Identifying biomarkers that predict TIL presence and activity can further enhance prognostic assessments and inform personalized treatment strategies. The therapeutic implications of TILs are profound, particularly in the context of immunotherapy. Immune checkpoint inhibitors, adoptive cell therapy, and combination treatments that integrate immunotherapy with conventional modalities show promise in enhancing antitumor responses.

## Author contributions

**Conceptualization:** Emmanuel Ifeanyi Obeagu.

**Methodology:** Emmanuel Ifeanyi Obeagu, Getrude Uzoma Obeagu.

**Resources:** Emmanuel Ifeanyi Obeagu.

**Supervision:** Emmanuel Ifeanyi Obeagu.

**Validation:** Emmanuel Ifeanyi Obeagu, Getrude Uzoma Obeagu.

**Visualization:** Emmanuel Ifeanyi Obeagu, Getrude Uzoma Obeagu.

**Writing – original draft:** Emmanuel Ifeanyi Obeagu, Getrude Uzoma Obeagu.

**Writing – review & editing:** Emmanuel Ifeanyi Obeagu, Getrude Uzoma Obeagu.
